# The Auditory Brainstem Response Diagnoses Alzheimer-Like Disease in the 5xFAD Mouse Model

**DOI:** 10.1523/ENEURO.0049-25.2025

**Published:** 2025-04-18

**Authors:** Daxiang Na, Yidan Yang, Li Xie, Dorota Piekna-Przybylska, Dominic Bunn, Maleelo Shamambo, Patricia White

**Affiliations:** ^1^Department of Neuroscience, Ernst J. Del Monte Institute for Neuroscience, University of Rochester Medical Center, Rochester, New York 14642; ^2^School of Mathematical Sciences, Rochester Institute of Technology, Rochester, New York 14623; ^3^Department of Biomedical Genetics, University of Rochester, Rochester, New York 14642

**Keywords:** 5xFAD, Alzheimer’s disease, auditory brainstem response, central auditory hyperactivity, machine learning, neuroinflammation

## Abstract

Early and accurate diagnosis of Alzheimer's disease (AD) will be key for effective personalized treatment plans ( Cummings, 2023). Significant difficulties in auditory processing have been frequently reported in many patients with mild cognitive impairment, the prodromal form of AD ( Tarawneh et al., 2022), making it an outstanding candidate as AD diagnostic biomarker. However, the efficiency of diagnosis with this parameter has not been explored. Here we show that when male mice with amyloidosis begin to show memory decline, changes in the auditory brainstem response (ABR) to clicks enable the reliable diagnosis of disease using a machine learning algorithm. Interpretation of the machine learning diagnosis revealed that the upper levels of the auditory pathway, including the inferior colliculus, were the probable sources of the defects. Histological analyses show that in these locations, neuroinflammation and plaque deposition temporally correlate with behavioral changes consistent with memory loss. While these findings are tempered by the caveat that they derive from amyloidosis mice, we propose that ABR measurements be evaluated as an additional rapid, low-cost, noninvasive biomarker to assist the diagnostic testing of early-stage AD.

## Significance Statement

New disease-modifying treatments for Alzheimer's disease (AD) only work for a subset of patients and require precise disease staging. AD is highly correlated with both central auditory dysfunction and hearing loss, but these are not diagnostic. We found that characteristics of the passive auditory brainstem response test reliably diagnose the onset of memory decline in a mouse model of AD, correlating with the initiation of amyloid deposits and neuroinflammation in the upper auditory nuclei. We further highlight the role that one region, the inferior colliculus, plays in multisensory integration, speculating that dementia becomes evident when the plaque-burdened cortex is unable to compensate for the degradation of preconscious sensory processing.

## Introduction

Alzheimer’s disease (AD) imposes a terrible burden on patients, their families, and society. AD hallmarks include deposits of amyloid-β (Aβ), hyperphosphorylation of the axonal protein Tau (tau tangles), and neurodegeneration (Shen et al., 2020). Recently, monoclonal antibodies against Aβ were approved by the FDA as disease-modifying treatments for AD (Dunn et al., 2021). Notably, patients experienced a range of responsiveness during trials, from improvement to little effect (Wu et al., 2023). This variation could result from disease heterogeneity, imprecision in staging, or both. Augmenting current diagnostic criteria with more biomarkers will improve our understanding and future treatment of AD.

AD strongly correlates with both hearing loss and dysfunctional central auditory processing (Gates et al., 2011; Lin et al., 2011). Two upper auditory brainstem nuclei, the medial geniculate body (MGB) of the thalamus and the inferior colliculus (IC), reside near the hippocampus where AD hallmarks likely initiate (Osen et al., 2019). Indeed, both Aβ plaques and tau tangles are reproducibly present in the IC in deceased AD patients (Ohm and Braak, 1989; Sinha et al., 1993). For some patients with mild cognitive impairment (MCI), the transition to AD is preceded by dysfunctional central auditory processing (Gates et al., 2011; Swords et al., 2018). Moreover, some MCI patients exhibit auditory midbrain hyperactivity (Bidelman et al., 2017), measured by the frequency-following response which is thought to reflect a neurophonic potential generated in the IC (Sohmer et al., 1977; Coffey et al., 2019). Taken together, the evidence suggests a correlation between AD and central auditory hyperactivity. However, these correlations exist on a population level. There are many older individuals with normal cognition who also have hearing disorders, in part because central auditory hyperactivity is a frequent sequela to noise exposure and hearing loss (Schrode et al., 2018).

The auditory brainstem response (ABR) is a simplified electroencephalogram recording of preconscious event-related potentials in response to auditory stimuli. In the clinic, it is typically used to determine auditory thresholds in infants. ABR is a mature and relatively inexpensive technology, and collecting data from a single stimulus takes a few minutes. As the ABR records brain function, it may be useful in diagnosing changes prior to the onset of structural losses. Notably, studies of ABR click recordings in older adults using modern equipment report variation in relevant parts of the waveform that do not correlate with hearing loss (Maass et al., 2024). Here, we use recordings from single suprathreshold stimulus in conjunction with a machine learning algorithm to diagnose relative changes in the responses of nuclei in the auditory brainstem that distinguish mice with and without amyloidosis. We correlate these diagnostics to the onset of memory loss of a conditioned stimulus and with neuroinflammation in the auditory brainstem nuclei thought to generate the waveform.

## Materials and Methods

### Ethics approval

All experiments were performed under protocol number 2010-011: PI Patricia White, in compliance with the United States Department of Health and Human Services, and were reviewed by the University of Rochester’s Committee on Animal Resources.

### Mice

5xFAD mice (Oakley et al., 2006) on a congenic C57BL/6J background were obtained from the Jackson Laboratory (stock number 34848-JAX). We compensated for the known hearing loss deficit in C57BL/6J (Noben-Trauth et al., 2003) by breeding it to wild-type (WT) CBA/CaJ, to obtain heterozygote mutant mice and their WT littermates with a CBA/B6 hybrid background. To eliminate effects of amyloidosis on maternal care, we bred transgenic 5xFAD males to CBA/CaJ females. Mice were weaned between Postnatal Day (P)21 and P28. To ensure the consistency in genetic background, only F1 heterozygote 5xFAD males and their WT male littermates were used in this study. Females were not analyzed due to early auditory differences with WT littermates (Na et al., 2023). The genotyping of tail samples was performed by Transnetyx Automated PCR Genotyping Services (Transnetyx). Mice were housed in standard cages with up to four same-sex littermates on a 12 h light/dark cycle, with *ad libitum* access to food and water, ample nesting materials, and small hide-away enclosures. Using the NIOSH Sound Level Meter app, ambient noise in the mouse colony room was estimated at 70 dB, with interior cage levels centered around 58 dB. All animal protocols were approved in advance by the University Committee for Animal Research at the University of Rochester Medical Center. Unless otherwise specified, mice were killed by exposing them to CO_2_ until their breathing had ceased for 1 min, followed by decapitation.

### ABR

The ABR data and click thresholds used in this report were previously published in Na et al. (2023) and utilized a system with a Smart EP Universal Smart Box (Intelligent Hearing Systems) and an ED1 speaker (Tucker-Davis Technologies). Testing occurred between the hours of 9 A.M. and 4 P.M. Mice were anesthetized with a single intraperitoneal injection of ketamine (80 mg/kg animal weight, Hikma Pharmaceuticals) and acepromazine (3 mg/kg, VetOne) diluted into a sterile saline solution. They were placed in an anechoic chamber used for closed field auditory testing. Three sterilized fine subdermal electrodes were inserted under the skin, one at the vertex and one behind each pinna. An interaural probe coupled to the speaker outputs was placed at the opening of each mouse's left external auditory meatus. The ABRs used in the machine learning model were in response to 50 µs click stimuli. Stimulus amplitudes decreased in 5 dB steps from 75 dB sound pressure level (SPL) to 5 dB SPL. The averages of 512 sweeps were recorded for each amplitude. Responses were rejected if their peak to trough amplitude was greater than 31 µV at any time between 1.3 and 12.5 ms after stimulus presentation. Well-anesthetized mice typically had a 5–30% rejection rate. Click ABRs were performed immediately after full motionlessness was achieved, to provide the cleanest signal. A Quest Technologies portable sound level meter, Model 1900, was used to calibrate the apparatus <1 month prior to the beginning of the experiments.

ABR peaks and troughs were registered by a reviewer blinded to the experimental design and mouse genotypes as described in Na et al. (2023). Wave latency was defined as the difference between the auditory stimuli onset (0 ms) and the time of the peak apex (ms). Wave amplitude was defined as the difference between the peak apex and the following trough (μV). All five waves (I, II, III, IV, and V) were extracted in this study.

### Histology of the brain

Histological analysis was performed on brains of 5xFAD mice aged 3 months (3M), 6M, and 12M, and WT littermates aged 12M (*n* = 6 for each age and genotype). During deep anesthesia, mice underwent transcardial perfusion with saline and 4% paraformaldehyde (PFA). The brains were removed and postfixed for 2 h at 4°C in 4% PFA. Brains were embedded in agarose, and 80 µ sections were cut on Compresstome (Precision Instruments). Sections were collected and stored in 24-well plates in PBS at 4°C.

Sections containing the brain nuclei of interest were selected by comparison with the Mouse Brain Atlas (Franklin and Paxinos, 2019). Regions of the auditory cortex (AC), the MGB, the IC, the medial nucleus of the trapezoid body (MNTB), the superior olivary complex (SOC), and the cochlear nucleus (CN) were analyzed for potential correlates to auditory processing disorder, as described in Na et al. (2023). The following primary antibodies were used in this study: rabbit anti-ionized calcium–binding adaptor molecule 1 (Iba1; 1:2,000, Wako–Sigma-Aldrich), rabbit anti-GFAP (1:1,000, Novus Biologicals), rat anti-cell differentiation 68 (CD68; 1:500, Bio-Rad Laboratories), and 6E10 (1:3,000, BioLegend). Floating sections were incubated in primary antibodies diluted in 0.5% Trion X-100/PBS (PBST) with 5% donkey serum overnight at 4°C. They were washed three times for 30 min in PBST with gentle rocking, further incubated with fluorescently labeled secondary antibodies/reagents (Alexa Fluor 594 and Alexa Fluor 647, Jackson ImmunoResearch Laboratories; NeuroTrace 500/525, Thermo Fisher Scientific; all at 1:500) at room temperature for 2 h, and washed three times for 30 min in PBST. Tissues were mounted in Fluoromount-G (catalog #0100-01, SouthernBiotech) and coverslipped on microscope slides.

### Histology of the cochlea

Cochleae were dissected from killed mice at designated time points. The stapes was removed, and a small incision was made at the apical tip of the cochlea for proper fluid exchange during immersion fixation with 4% PFA diluted in PBS. Tissues were transferred to decalcifying 0.1 M ethylenediaminetetraacetic acid at 4°C on a rocker for 3 d.

For cryosectioning, tissues were submerged in 30% sucrose/1× PBS overnight, embedded in optimal cutting temperature (OCT) compound (Sakura Finetek), and frozen with liquid nitrogen. These tissues were sectioned at 20 μm with the 16 and 32 kHz regions visible and mounted onto slides (Fisherbrand Superfrost Plus, Thermo Fisher Scientific). The 16 and 32 kHz regions were selected to analyze changes.

For immunohistochemical staining on cryosections, sections were washed with PBST at pH 7.4 and then blocked for 1 h at room temperature in 5% normal donkey serum diluted in PBST. Antigen retrieval was performed prior to the immunostainings by boiling slides in 10 mM citric acid, pH 6, for 15 min at 20% microwave power. Antibody incubations were performed overnight at 4°C in blocking solution. The following primary antibodies were used in this analysis: mouse TUJ1 (anti-beta Tubulin 3, 1:100, R&D Systems) and mouse anti- calbindin 2 (Calb2, 1:100, Millipore Sigma). Sections were washed in PBST at room temperature prior to secondary antibody incubation overnight at 4°C in the dark. Alexa Fluor-conjugated secondary antibodies included 488, 594, and 647 (Thermo Fisher Scientific) and were diluted at 1:500 each. A 4′6-diamidino-2-phenylindole (DAPI) diluted at 1:10,000 was added during the secondary antibody incubation. Sections were washed in PBST and mounted in Fluoromount-G (SouthernBiotech) with coverslips.

For the synapse analysis, whole-mount preparation was performed following cochlear dissection and decalcification. Cochleae were microdissected into three turns (apical, middle, and basal) as previously described (Montgomery and Cox, 2016). These pieces were frequency mapped using the ImageJ 64 (NIH) plugin from Massachusetts Eye and Ear Infirmary and immunostained for quantification. Antigen retrieval was performed by snap-freezing in liquid nitrogen, followed by thawing at room temperature for 1 h. The following primary antibodies were used in this analysis: mouse anti-C-terminal binding protein 2 (anti-CtBP2, 1:200, BD Biosciences), rabbit anti-vesicular acetylcholine transporter (anti-VAT, 1:500, Millipore Sigma), and goat anti-oncomodulin (anti-OCM, 1:500, Thermo Fisher Scientific).

### Behavioral assays

Conditioned fear responses are impaired in patients with mild to moderate AD (Hamann et al., 2002), suggesting this paradigm for measuring cognitive changes. Mice were tested with open-field testing (Day 1) and fear conditioning (Day 3). Fourteen days before behavioral testing, mice were switched to a reverse light/dark cycle room. For 3 d before behavioral testing, mice were transported from the colony room to the behavior room, handled for 5 min, and then returned to the colony room within the same day. All experiments were carried out between 9 A.M. and 5 P.M. 5xFAD mice and their WT littermates within each cohort were tested on the same day for each experiment at each age. The same experimenter performed all the behavioral tests presented in this study and was blinded to genotype. The tests were carried out as described in Owlett et al. (2022) with modifications described below.

#### Open-field testing

Open-field testing was performed to measure anxiety and locomotor function. Each mouse was allowed to *ad libitum* explore within a 31 × 31 cm box for 5 min. The periphery of the box was defined as 5 cm from the box edges, and the center zone was defined as the remaining internal area. Distance (meters) and head entries into the center zone were measured automatically by the ANY-maze software (Stoelting). The center zone entry was calculated by normalizing the total number of head entries into the center zone with the total distance traveled.

#### Fear conditioning

Cued and contextual fear conditioning was used to assess conditioned memory. On the conditioning day, each mouse was allowed to *ad libitum* explore the conditioning chamber which consisted of a Plexiglass chamber and metal floor grid (Coulbourn Instruments). After 3 min, they were given three footshocks (0.5 mA for 2 s each) during the coterminal presentation of white noise at 80 dB SPL for 15 s. Twenty-four hours later, the mouse was placed back into the same chamber, and its freezing responses were measured for 5 min to test its contextual long-term memory (familiar context session, referred to as “familiar”). Four hours later, the mouse was placed in a novel context environment consisting of a 15 cm open-topped plastic cylinder with bedding on the floor for 3 min (novel context session, referred to as “novel”) followed by re-exposure to the white noise for 3 min, to test hippocampal-independent memory (cue test, referred to as “cue”). The proportion of time that the animal was frozen during the first 30 s freezing time was multiplied by 100 to calculate the freezing score in the familiar context, novel context, and tone test sessions. All data were video recorded and scored using the ANY-maze software.

### Machine learning classification

#### Feature extraction

The ABRs evoked by 65 dB SPL click stimuli were selected for analysis, as this sound level was above the hearing threshold for all mice tested. Click stimuli were utilized because they activate a broad frequency response for assessing auditory pathway integrity and because all five waves were consistently present among all mice. ABRs were recorded from 5xFAD mice and their WT littermates at 3, 6, and 12M. Eighteen variables were extracted from those ABRs and referred to as “features” in the machine learning classification, including Wave I (p1) amplitude and latency, Wave II (p2) amplitude and latency, Wave II to I amplitude ratio (p2:p1 ratio), Wave I to II latency interval (p2–p1 interval), Wave III (p3) amplitude and latency, Wave III to I amplitude ratio (p3:p1 ratio), Wave I to III latency interval (p3–p1 interval), Wave IV (p4) amplitude and latency, Wave IV to I amplitude ratio (p4:p1 ratio), Wave I to IV latency interval (p4–p1 interval), Wave V (p5) amplitude and latency, Wave V to I amplitude ratio (p5:p1 ratio), and Wave I to V latency interval (p5–p1 interval).

#### Machine learning classification and cross-validation

ABRs were used to diagnose disease in 5xFAD mice with machine learning classification. Machine learning classification and feature-importance analysis were carried out with the open-source CatBoost model (Prokhorenkova et al., 2018). In cross-validation, data of all 18 variables of the ABR data mentioned above were fed into the model for training or computing outcome; the genotypes of the mice (WT or AD) from which the ABRs were recorded were used for training or validation. Classification experiments were implemented using stratified threefold cross-validation to preserve the same percentage of samples for each class to improve robustness. In this step, the dataset was partitioned into three equal-sized subsamples (folds). A single subsample of the threefold was retained as the validation data for model testing; the remaining two subsamples were used as training data. Each subsample was classified by the machine learning model trained from a dataset not including this subsample itself; therefore a generalized estimation of the classification performance was achieved. The total outcome of the cross-validation was then used to calculate the ROC curve, accuracy, sensitivity, and specificity.

Feature-importance analysis calculates a score for the contribution of all the input features to the classification. In the present study, feature-importance values were normalized so that the sum of importances of all features was equal to 100 (therefore the numbers are presented as percentages). A higher score of feature importance indicated that this feature contributed more to the disease identification in our analysis and therefore indicated a more pronounced difference between WT and AD mice in this variable.

#### ROC and AUC

The area under the receiver operating characteristic (ROC) curve (AUC) was calculated to evaluate the performance of classification on ABRs at different ages. The ROC curve was a plot of the true-positive rate versus the false-positive rate of a classification task. The different points on the curve corresponded to the different criteria of probability used to determine whether the genotype was AD or WT. A classification result that was better than a random classifier would reveal an ROC curve above the diagonal line on the ROC curve plot. The AUC, which was computed as the area under the ROC curve, is commonly used as an effective way to summarize the overall performance of the classification (Mandrekar, 2010). In general, an AUC of 0.5 suggested no discrimination (i.e., no ability to diagnose patients/subjects with and without the disease based on the test), 0.7 to 0.8 was considered acceptable, 0.8 to 0.9 was considered excellent, and >0.9 was considered outstanding for a clinical diagnostic test (Mandrekar, 2010).

#### Accuracy, sensitivity, and specificity

Accuracy, sensitivity, and specificity are commonly used to evaluate the performance of prediction and diagnosis. Accuracy was calculated by the total number of correctly classified subjects divided by the total number of subjects. The sensitivity was calculated by the total number of correctly classified positive subjects divided by the total number of positive subjects. Specificity was calculated by the total number of correctly classified negative subjects (WT mice) divided by the total number of negative subjects.

The cross-validation, ROC plotting, and AUC calculation were performed with the built-in functions in the Python scikit-learn package (Buitinck et al., 2013).

### Experimental design and statistical analysis

5xFAD is a commonly used amyloidosis model for AD which develops hearing loss and central auditory hyperactivity, measured with the ABR response, during disease progression. In this study we wanted to determine if machine learning could be used to diagnose amyloidosis from the ABR data. We further examined the timing of when differences in auditory responses arose compared with performance in memory-dependent assays and if the diagnostic features of the ABR waveform correlated with neuroinflammation in their neural generators. Lastly, we examined the cochlea of 5xFAD mice for pathology that could explain the results. Experiments are listed in order of presentation in the Results section. Only male mice were used, due to early developmental differences in the auditory system of 5xFAD female mice prior to amyloid accumulation (Na et al., 2023). The researcher was blinded to the mouse genotypes, ages, and conditions for all analyses. The Shapiro–Wilk test was used to assess normality for each dataset, and nonparametric tests were used for all non-normal datasets.

#### Open-field and fear conditioning

Three sets of mice of different ages were each tested one time. For open-field testing, total distance traveled in meters and the number of head entries into the center zone were both recorded. Center zone entry was calculated by normalizing the total number of head entries into the center zone with the total distance traveled. The same mice were tested with fear conditioning. The percentage of time spent motionless in the first 30 s after being placed in a familiar or novel environment, or after cue presentation, was recorded. We tested 14 WT and 10 5xFAD males aged 3M; 14 WT and 8 5xFAD males aged 6M; and 12 WT and 11 5xFAD males aged 12M. Two-way ANOVA and unpaired Student's *t* test with the Bonferroni’s adjustment were used to assess differences in the open field, while the Wilcoxon test was used to assess differences in fear conditioning.

#### Machine learning analysis

One ABR waveform of a click stimulus presented at 65 dB was used from each mouse. This stimulus was chosen because it was within the hearing thresholds of all mice, generated well-defined waveforms, and required no assumptions about frequency-specific processing. We used data from 9 WT and 14 5xFAD mice at 3M; 21 WT and 25 5xFAD mice at 6M; and 21 WT and 15 5xFAD mice at 12M.

#### Neuroinflammation histological analyses

For each mouse used, 3–4 coronal sections containing the AC, MGB, IC, CN, SOC, and MNTB were selected by comparison with the Mouse Brain Atlas for staining, imaging, and quantification. Sections were imaged with a Leica Stellaris 5 Inverted Confocal Microscope (Leica Microsystems) using a 10× objective. Imaging parameters were adjusted to minimize signal saturation and kept constant across all sections for each set of immunofluorescent labels. All images were processed with ImageJ Fiji (NIH) and analyzed with a custom CellProfiler (Stirling et al., 2021) pipeline.

For quantification of the total area covered by microglia or activated astrocytes, regions of interest (ROIs) outlining the abovementioned structures were drawn on maximum *z*-projections of the acquired images, and the corresponding masks were generated with a custom ImageJ plugin. Images were subsequently thresholded and binarized using the “Otsu” thresholding algorithm embedded in CellProfiler. The microglia and activated astrocytes were calculated as the ratio between the number of pixels thresholded over all pixels in the ROIs. In the present study, microglia coverage is presented as “% Microglia Coverage (Iba1),” and activated astrocyte coverage is presented as “GFAP Area Fraction (%)”. For quantification of microglia activation, the CD68 images were thresholded and binarized in the same regions, and the overlap between CD68 and microglia was measured by the “RelatedObjects” function. The number of colocalized signal pixels was divided by the total microglia pixels to calculate the fraction of CD68-expressing microglia. We compared six 5xFAD mice at each of the three ages (3, 6, and 12M) to six WT mice aged 12M, using Wilcoxon with the Holm–Šídák multiple-comparison test.

#### Cochlear histology

For spiral ganglion neuron (SGN) analysis, four 5xFAD and four WT cochleae, both from 12M mice, were sectioned to reveal the 16 and 32 kHz turns; stained for Calb2, TuJ1, and DAPI; and imaged at 40×. Sections were imaged using an Olympus FV1000 (Olympus) laser scanning confocal microscope. For SGN analysis, images were acquired with five *z*-stacks which were centered at the sagittal plane of the section. The *z*-projection of the stacks was used for quantification. TUJ1-positive and Calb2-positive cells were counted by an experimenter blinded to animal information with the built-in cell counting function in ImageJ Fiji (NIH). The Type 1 SGN density was calculated by dividing the TUJ1-positive cell number by the area of the spiral ganglion region. Type 1 SGNs include three subtypes: Types 1a and 1b are Calb2-positive cells, while Type 1c is Calb2-negative cells (Shrestha et al., 2018). The Calb2-negative cell number was calculated by subtracting the TUJ1-positive cell number from the Calb2-positive cell number, then dividing by the TUJ1-positive cell number to calculate the proportion of Type 1c cells.

To assess the synapse loss in 5xFAD cochleae, the 8, 16, and 32 kHz regions from the whole mounts were imaged at 100× on the Olympus FV1000 microscope. The frequencies were selected given that significant hearing loss was present at those frequencies (Na et al., 2023). Images were converted (.oif to .ims) and imported into the Imaris 9.3 Image Visualization and Analysis Software (Oxford Instruments) for 3-D reconstruction. CtBP2 and VAT isosurfaces in inner cell regions and VAT isosurfaces in outer cell regions were thresholded and constructed. The number of CtBP2 isosurfaces and the volume of VAT isosurfaces were calculated with the Imaris built-in function. The inner hair cell (IHC) nuclei were identified by the CtBP2 staining with the shape distinctive from ribbon synapses. The outer hair cells (OHCs) were identified with OCM staining.

A *p* < 0.05 was considered statistically significant. Data are presented as modified box or bar plots (with error bars representing SEM). For all plots, individual data are presented as dots. In all figures, the *p* values are defined as follows: no significance (ns); *p* ≥ 0.05; *p < 0.05; **p < 0.01; and ***p < 0.001 unless otherwise noted. Statistics and data plotting were performed in GraphPad Prism 9.3.1 and R 4.1.2.

## Results

In a previous study, we tested two strains of amyloidosis mice for auditory dysfunction, including hearing loss, hearing in noise, and central auditory hyperactivity (Na et al., 2023). Two findings were key. First, we observed both hearing loss and central auditory hyperactivity in 5xFAD mice, where Aβ accumulated in the MGB and IC and, to a lesser extent, the SOC. Second, donepezil administration to 5xFAD mice reversed central auditory hyperactivity. Donepezil is an acetylcholinesterase inhibitor commonly prescribed to MCI and early AD patients. Neuroinflammation has been proposed to link AD and hyperactivity (Hector and Brouillette, 2020). However, the status of the inflammatory response, including the microglia and astrocyte activity, in the auditory brainstem of amyloidosis mice remains undetermined. Lastly, the onset of memory deficits has not been temporally related to that of central auditory hyperactivity in the mouse model.

### Onset of behavioral changes in 5xFAD mice on the CBA/CaJ background

**Figure 1.**
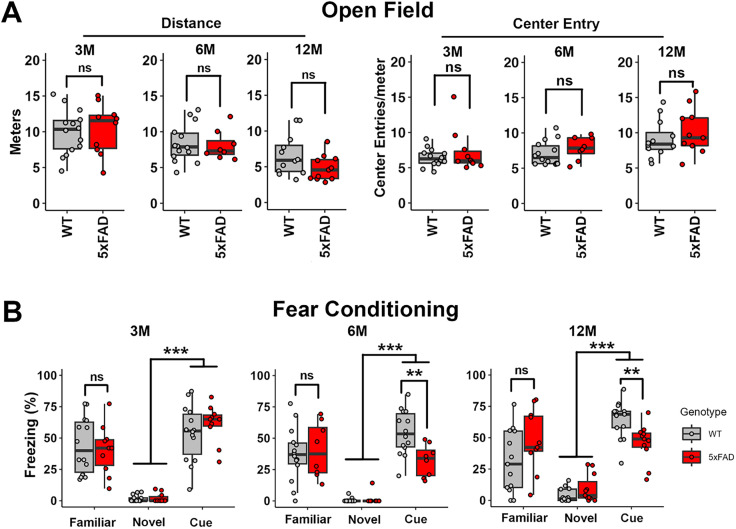
5xFAD mice first exhibit reduced fear responses to a loud paired stimulus at 6M. ***A***, Quantification of open-field test performance, with total traveling distance (Distance, left panel) and tendency to explore the center zone (Center Entry, right panel) scored. No differences in motility or anxiety-related behaviors between genotypes are observed. ***B***, Quantification of cued and contextual fear conditioning test performance in 5xFAD mice and their WT littermates at 3, 6, and 12M (3M, WT, *n* = 14; 5xFAD, *n* = 10; 6M, WT, *n* = 14; 5xFAD, *n* = 8; 12M, WT, *n* = 12; 5xFAD, *n* = 11). Scores in the familiar context session (Familiar), novel context session (Novel), and cue test session (Cue) are shown. 5xFAD mice have a significantly reduced response to the cue, beginning at 6M. Asterisks denote the significance of differences between WT and 5xFAD mice: no significance (ns), *p* ≥ 0.05; *p < 0.05; **p < 0.01; and ***p < 0.001; Student's *t* test, Wilcoxon test, or two-way ANOVA.

To establish when amyloidosis induces memory loss for the test subjects, we performed the open-field test and the cued fear conditioning test on WT and 5xFAD mice at 3, 6, and 12M. Open-field testing enabled the evaluation of locomotor activity and anxiety levels of 5xFAD mice, an important control for subsequent tests that assess freezing behavior. Locomotor activity was evaluated by scoring the total distance traveled during the test session (Fig. 1*A*, left graphs). 5xFAD mice did not perform significantly worse than WT littermates at any stage (3M, *p* = 0.76; 6M, *p* = 0.82; 12M, *p* = 0.079; unpaired Student's *t* test and Wilcoxon test). The center entry was scored to evaluate the tendency of exploring the center zone. A reduced anxiety level has been correlated to an increased tendency to explore the center zone (Kraeuter et al., 2019). 5xFAD mice did not show significant changes in center entry at any stage compared with WT littermates (3M, *p* = 0.93; 6M, *p* = 0.30; 12M, *p* = 0.41; Student's *t* test or Wilcoxon test; Fig. 1*A*, right graphs).

The cued fear conditioning test is commonly used to evaluate memory (Curzon et al., 2009). The freezing score in the novel context and cue test sessions were examined to evaluate each mouse's performance following fear conditioning. Note that cues are presented at 80 dB, which is greater than the mouse auditory thresholds for both genotypes at all stages (Na et al., 2023). The session difference between novel context and cue test sessions was significant for mice at 3, 6, and 12M, suggesting that the training was successful (3, 6, and 12M, *p* < 0.001; two-way ANOVA; Fig. 1*B*). 5xFAD mice performed worse in the cue test at 6 and 12M compared with their WT littermates (3M, *p* = 0.39; 6M, *p* = 0.0002; 12M, *p* = 0.0016; two-way ANOVA followed by Bonferroni’s post hoc analysis; Fig. 1*B*, middle and right graphs). The freezing score in the familiar context session was examined to evaluate contextual long-term memory in 5xFAD mice at 3, 6, and 12M (Fig. 1*B*). 5xFAD mice did not show a significantly worse performance at any stage compared with their WT littermates in this part of the evaluation (3M, *p* = 0.69; 6M, *p* = 0.76; 12M, *p* = 0.11; Wilcoxon test). Overall, we detected a significant behavioral change in 5xFAD mice beginning at 6M in the cued fear conditioning test. Notably, this correlates with the onset of a statistically significant increase in central auditory hyperactivity but precedes hearing loss in this mouse line (Na et al., 2023).

### A machine learning model can diagnose amyloidosis status in male mice from the click ABR responses

**Figure 2.**
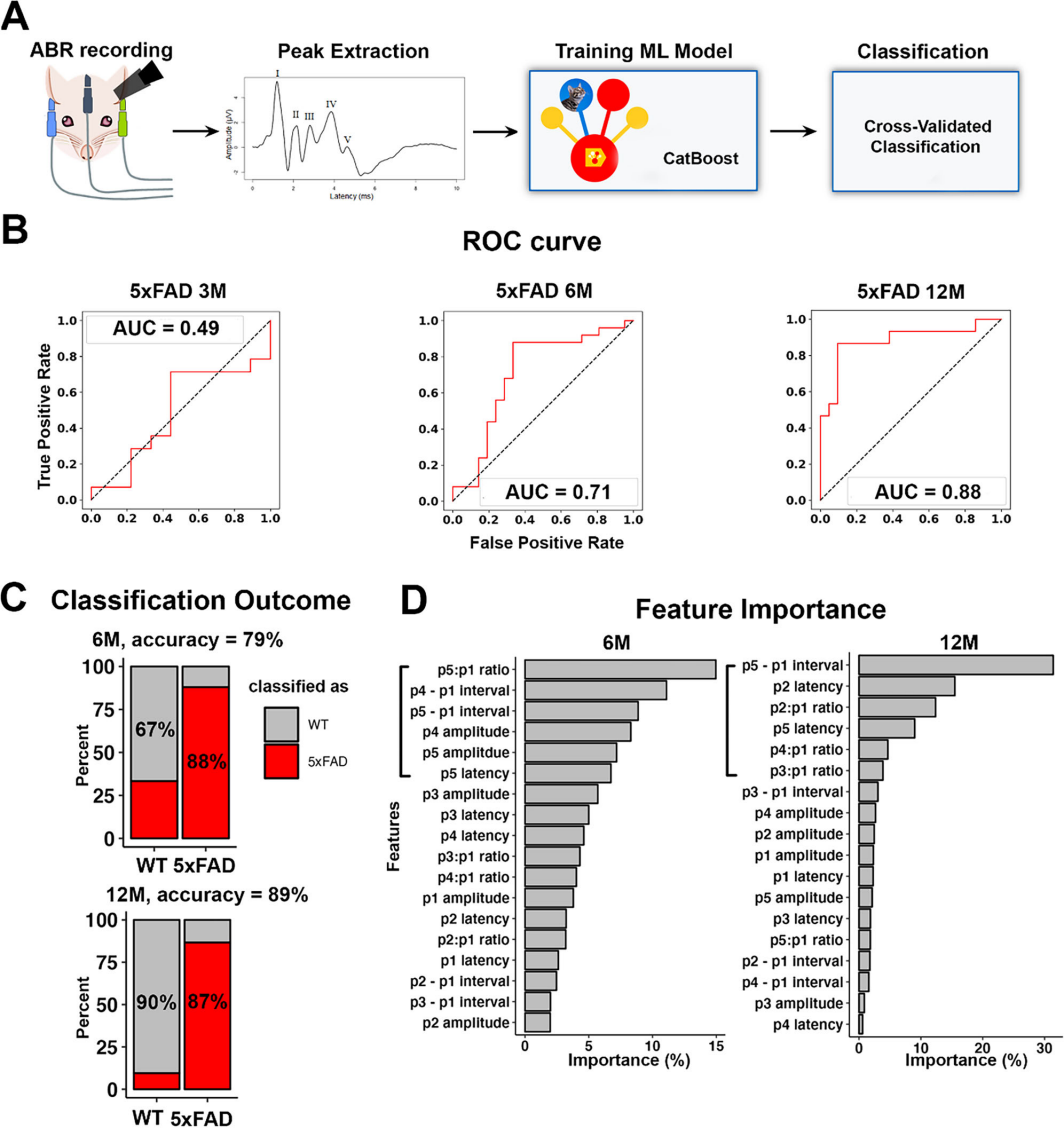
A machine learning algorithm can reliably identify 5xFAD mice based on differences in the later parts of their ABR waveforms, beginning at 6M. ***A***, The workflow of machine learning classification. ***B***, ROC curves (red) for the classification of ABRs from mice at 3M (WT, *n* = 9; 5xFAD, *n* = 14), 6M (WT, *n* = 21; 5xFAD, *n* = 25), and 12M (WT, *n* = 21; 5xFAD, *n* = 15). ***C***, Bar plots of the confusion matrix with the percentage of correctly classified objects in each genotype, i.e., the sensitivity and specificity of the classification, for WT and 5xFAD mice at 6M (top) and 12M (bottom). ***D***, The feature-importance maps of the classification for ABRs (left, 6M 5xFAD vs WT; right, 12–13M 5xFAD vs WT). The left square bracket highlights the top six features contributing to the classification of ABR into WT and 5xFAD genotypes.

We previously showed that multiple aspects of central auditory responses significantly altered with age in 5xFAD mice, suggesting that serial testing of central auditory activity could be an AD biomarker (Na et al., 2023). To evaluate whether amyloidosis could be diagnosed from the ABR waveforms and also to acquire a deeper understanding about the auditory brainstem activity changes induced by AD, we carried out machine learning classification on ABRs from mice at 3, 6, and 12–13M. Figure 2*A* shows the workflow for the classification: clicked-evoked ABRs were recorded from 5xFAD mice and their WT littermates at different ages as indicated in Figure 2*B*. The first five ABR waves were identified after recording, and their amplitude and latency were extracted. The ABR data were then fed into the CatBoost model, and ABRs were classified as WT or AD by the model after training as described in Materials and Methods. Figure 2*B* shows the ROC curves for the cross-validated classification. For the classification with ABR data from 5xFAD mice and their WT littermates at 3M, the AUC of the ROC curve was 0.49, suggesting that the classification is not better than a random classifier (an AUC score of 0.5 was expected from classification by chance alone). For the classification with ABR data from 5xFAD mice and their WT littermates at 6M, the AUC of the ROC curve was 0.71 (Fig. 2*B*), which is generally considered acceptable (Mandrekar, 2010). The classification yielded 79% accuracy (with 88% sensitivity and 67% specificity; Fig. 2*C*, top panel), which is comparable to the clinical diagnosis for AD (Beach et al., 2012). For the classification with ABR data from mice at 12M, the AUC of the ROC curve further increased to 0.88 for the 5xFAD mouse prediction, with improved accuracy (89%), comparable sensitivity (87%), and improved specificity (90%; Fig. 2*B*,*C*, bottom panel). These results suggest that the prediction performance improved along with disease progression.

Given that the machine learning classification revealed reliable results for 5xFAD mice at 6 and 12M, we further interpreted the classification by extracting the feature importance. For the classification on mice at 6M, we found that the top six features, which together accounted for >50% of the total importance (57%), were exclusively from Waves IV and V (Fig. 2*D*, left). For the classification of mice at 12M, the top six features were from the waves following Wave I (Waves II, III, IV, and V; Fig. 2*D*, right). While the latency or peak intervals reflect the conduction velocity of the pathway, the amplitude or amplitude ratio between Waves II–V and Wave I reflected the activity changes of the ABR neural generators (Eggermont, 2019). ABR Waves IV and V are suggested to be generated by regions including the IC and the SOC, while Waves II and III are proposed to be generated by the CN and SOC (Melcher et al., 1996; Melcher and Kiang, 1996; Land et al., 2016; Kim et al., 2022). Therefore, the feature-importance map suggested that in 5xFAD mice, the activity change may occur in the higher levels of the auditory pathway, such as the IC or SOC of 5xFAD mice, early in the disease, then extend to the entire central auditory pathway along with the disease progression. We had previously showed that among ABR generators, significant amounts of Aβ plaque were present in the IC, MGB, and SOC of 5xFAD mice beginning at 6M (Na et al., 2023), which is consistent with the feature-importance map.

### Neuroinflammation in the upper levels of the auditory pathway in 5xFAD mice begins at 6M of age

Multiple lines of evidence suggested that neuroinflammation might be the link between AD and the central auditory hyperactivity in 5xFAD mice. First, we previously performed a comprehensive examination on Aβ plaque deposition in the central auditory system of the 5xFAD mice and found that Aβ plaque was prominent in the upper levels of auditory pathway in 5xFAD mice (Na et al., 2023). Neuroinflammation, which is typically characterized by microglia and astrocyte activation, has been suggested as the downstream effect of plaque deposition; therefore, it is likely to occur along with the Aβ plaque observed in our 5xFAD mice (Long and Holtzman, 2019; Leng and Edison, 2021). This pattern of plaque distribution is consistent with the activity change pattern suggested by the machine learning classification (Fig. 2*D*; Na et al., 2023). Notably, work by other groups has suggested that neuroinflammation is involved in mediating the excitation-to-inhibition imbalance in the auditory system (Wang et al., 2019; Shulman et al., 2021), which is a potential cause for the central hyperactivity we observed in our 5xFAD mice (Na et al., 2023). Therefore, to further examine the correlation between AD and central auditory hyperactivity, we assessed the microglia and astrocyte activation in the central auditory pathway of AD mice.

**Figure 3.**
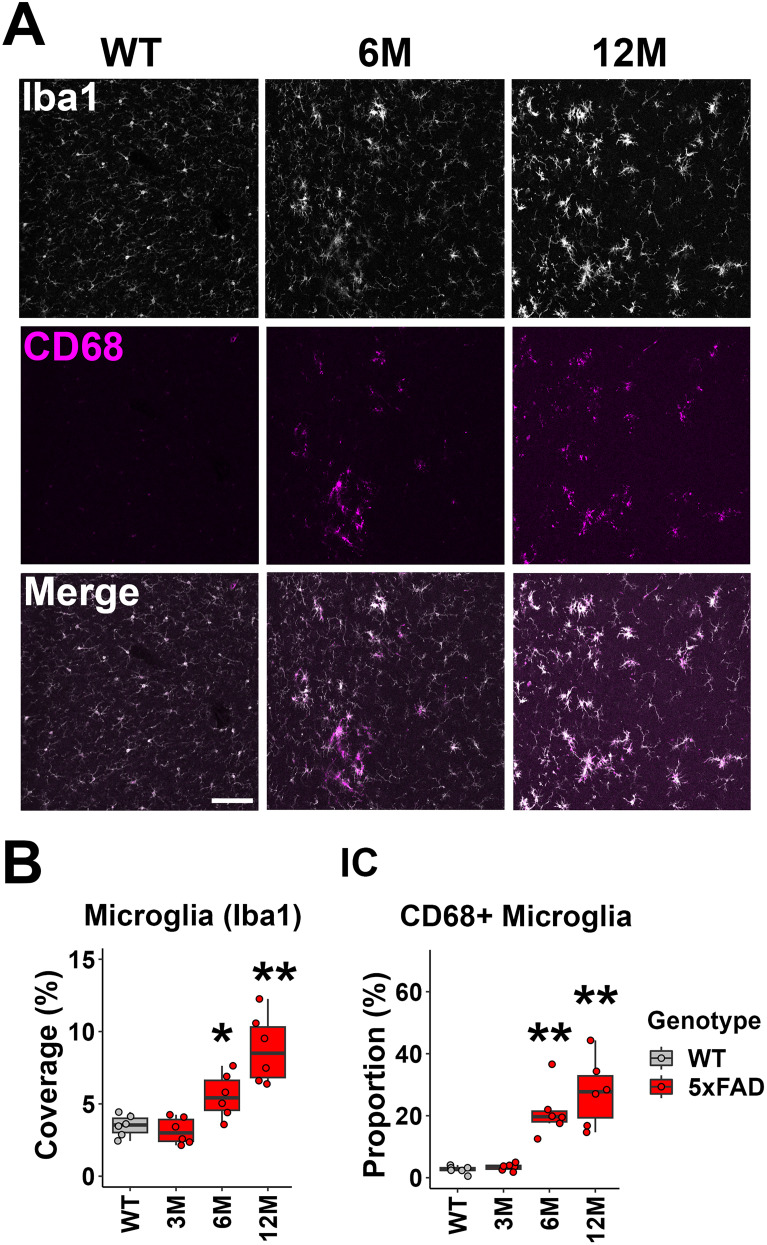
6M old 5xFAD mice exhibited neuroinflammation associated with microglia activation in the IC, which contributes to the later ABR waveform. ***A***, Representative views of the IC from WT mice at 12M and 5xFAD mice at 6 and 12M. The top row shows Iba1 immunostaining (white), the middle row shows CD68 immunostaining (magenta), and the bottom row shows the merged image. Scale bar, 100 μm. ***B***, The percentage of the area covered by microglia (left) and the proportion of CD68+ microglia (right) are displayed (*n* = 6 for each group). Data are presented by modified box plots with jitter points to represent individual animals. Asterisks denote the significant differences between WT and 5xFAD mice: no significance (no asterisk), *p* ≥ 0.05; *p < 0.05; **p < 0.01; and ***p < 0.001.

We evaluated microglia density using immunohistochemistry on brain sections. The microglia-specific marker, Iba1, was used to characterize the density of microglia in ROIs. In the IC, significant increases in microglia density started at 6M for 5xFAD mice (mean, 5.56%; *p* = 0.026; Wilcoxon test) and further increased by 12M (mean, 8.80%; *p* = 0.002; Wilcoxon test; Fig. 3*A*, top panel; *B*, left graph). CD68 was labeled in brain sections to characterize phagocytic activity of microglia (Hopperton et al., 2018). The proportion of microglia coexpressing CD68 increased significantly in the IC of 5xFAD mice at 6M (mean, 1.23%; *p* = 0.002; Wilcoxon test; Fig. 3*A* middle panel; *B* right graph), consistent with our previous observation of Aβ plaque deposition in this region (Na et al., 2023). It further increased in these mice at 12M (mean, 2.46%; *p* = 0.002; Wilcoxon test; Fig. 3*A*, middle panel; *B*, right graph).

**Figure 4.**
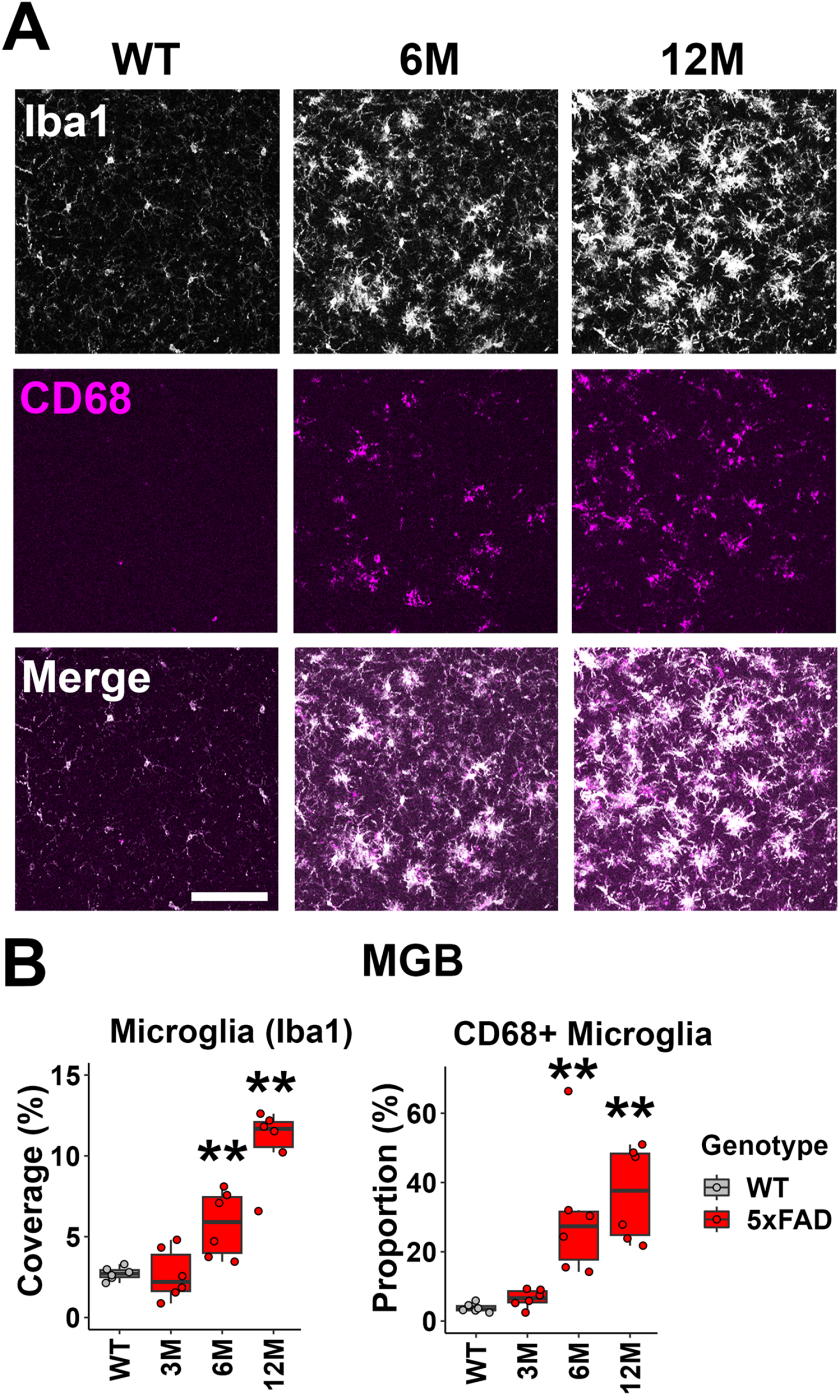
6M old 5xFAD mice also exhibited neuroinflammation associated with microglia activation in the MGB, which contributes to the later ABR waveform. ***A***, Representative views of the MGB from WT mice at 12M and 5xFAD mice at 6 and 12M. The top row shows Iba1 immunostaining (white), the middle row shows CD68 immunostaining (magenta), and the bottom row shows the merged image. Scale bar, 100 μm. ***B***, The percentage of the area covered by microglia (left) and the proportion of CD68+ microglia (right) are displayed (*n* = 6 for each group). Data are presented by modified box plots with jitter points representing individual animals. Asterisks denote the significant differences between WT and 5xFAD mice: no significance (no asterisk), *p* ≥ 0.05; *p < 0.05; **p < 0.01; and ***p < 0.001.

**Figure 5.**
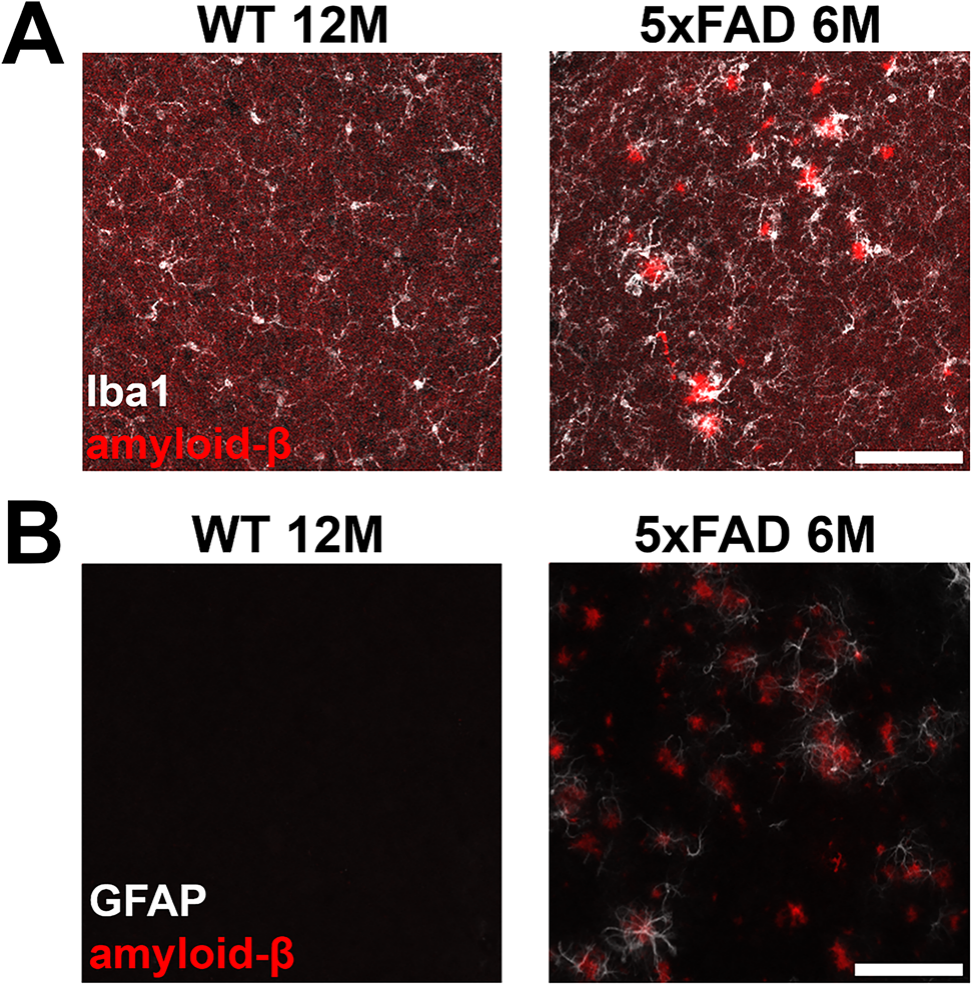
Microglia and reactive astrocytes are associated with amyloid plaque in the upper auditory brainstem of 5xFAD mice. ***A***, Representative images of Iba1 (white) and Aβ (red) staining in the IC of WT mice (12M) and 5xFAD mice (6M). ***B***, Representative images of GFAP (white) and Aβ (red) staining in the IC of WT mice (12M) and 5xFAD mice (6M). Scale bar, 100 μm.

The microglia density (Iba1+ cells) and proportion of CD68+ microglia also showed a significant increase in the MGB of 5xFAD mice beginning at 6M (microglia coverage: 6M 5xFAD, *p* = 0.002; 12M 5xFAD, *p* = 0.002; Wilcoxon test; CD68+ microglia proportion: 6M 5xFAD, *p* = 0.002; 12M 5xFAD, *p* = 0.002; Wilcoxon test; Fig. 4). Given that both the density and phagocytic activity of microglia showed significant increases in the IC and MGB of 5xFAD mice, we conclude that microglia activation was significant in the upper auditory brainstem of 5xFAD mice beginning at 6M. We further determined that both activated microglia and reactive astrocytes showed obvious association with amyloid plaque in the auditory brainstem of 5xFAD mice (Fig. 5), suggesting reactive gliosis as the downstream effect of plaque deposition.

### Microglia activation in the AC of 5xFAD mice

**Figure 6.**
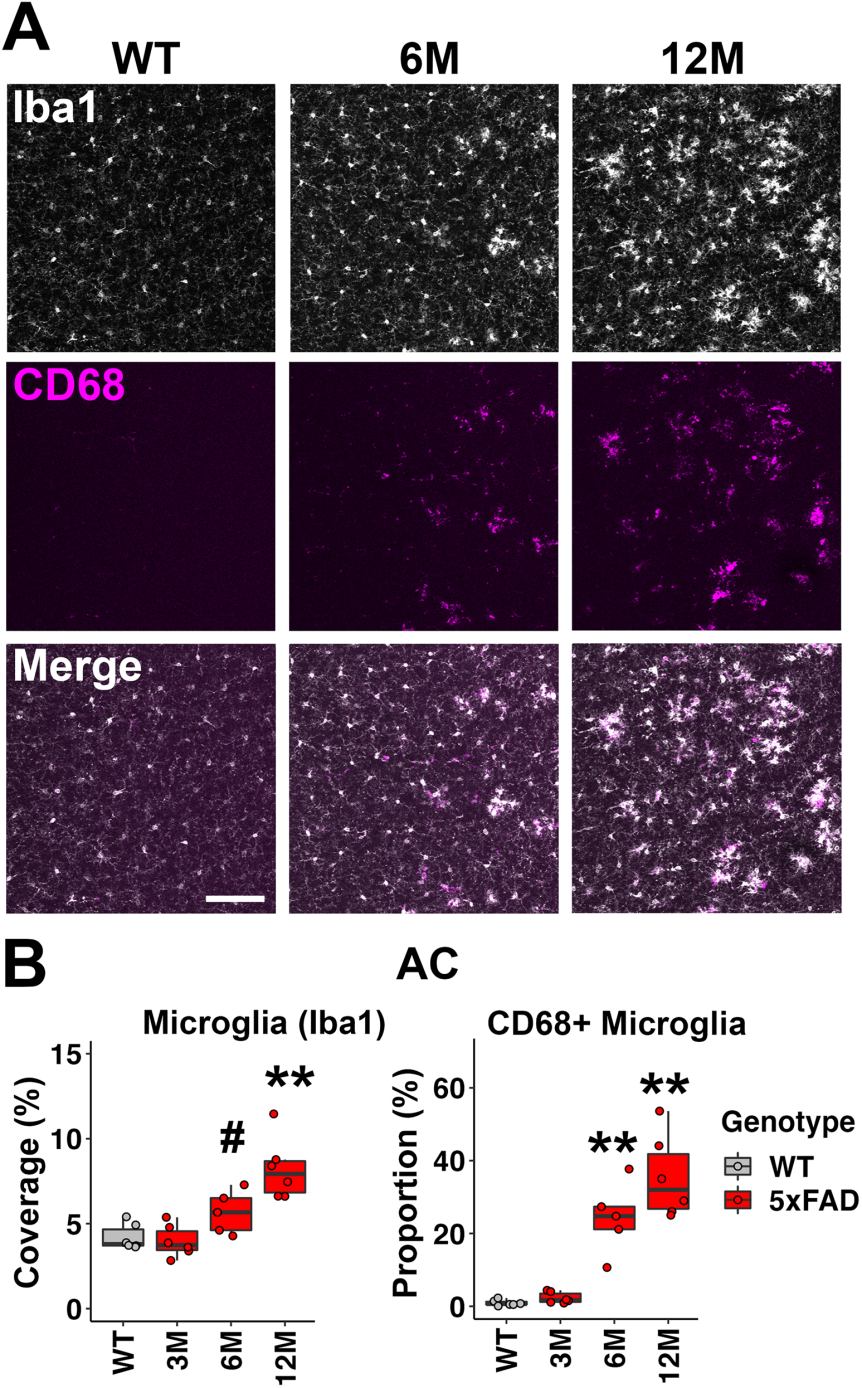
Neuroinflammation associated with microglia activation was also prominent in the AC of 6M old 5xFAD mice. ***A***, Representative views of the AC from WT mice at 12M and 5xFAD mice at 6 and 12M. The top row shows Iba1 immunostaining (white), the middle row shows CD68 immunostaining (magenta), and the bottom row shows the merged image. Scale bar, 100 μm. ***B***, The percentage of the area covered by microglia (left) and the proportion of CD68+ microglia (right) are displayed (*n* = 6 for each group). Data are presented by modified box plots with jitter points representing individual animals. Asterisks denote the significant differences between WT and 5xFAD mice: no significance (no symbol), *p* ≥ 0.05; ^#^*p* = 0.052; *p < 0.05; **p < 0.01; and ***p < 0.001.

Although not proposed as an ABR generator, the AC potentially regulates the activity of multiple levels of the auditory brainstem via corticofugal projections (Terreros and Delano, 2015). We therefore examined microglia activation in the AC. For 5xFAD mice at 6M, the microglia density showed an increasing trend, but the difference was not significant (mean, 5.67%; *p* = 0.052; Wilcoxon test; Fig. 6*A*,*B*, left graph); it further increased in mice at 12M and became significant (mean, 8.22%; *p* = 0.002; Wilcoxon test; Fig. 6*A*,*B*, left graph). An increase of microglial phagocytic activity was evident in the AC of 5xFAD mice at 6 and 12M (6M, *p* = 0.004; 12M, *p* = 0.002; Wilcoxon test, Fig. 6*A*,*B* right graph). Taken together, those analyses show that microglia activation was evident in the AC of 5xFAD mice at 6M.

### Astrocyte activation was prominent in the upper auditory brainstem and cortex of 5xFAD mice beginning at 6M of age

**Figure 7.**
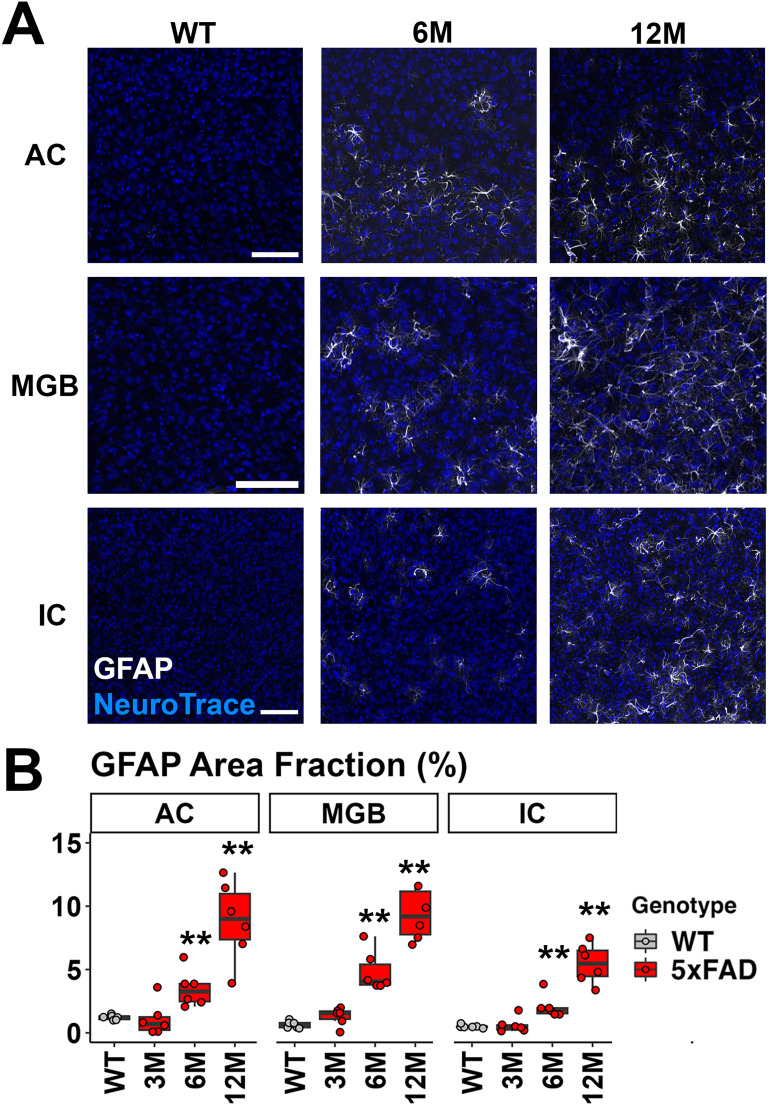
Neuroinflammation associated with astrocyte activation was also prominent in 5xFAD mice beginning at 6M in the AC and upper auditory brainstem. ***A***, Representative views of the AC, MGB, and IC from WT mice at 12M and 5xFAD mice at 6 and 12M. Scale bar, 100 μm. ***B***, The percentage of the area covered by GFAP is displayed (*n* = 6 for each group). Data are presented by modified box plots with jitter points representing individual animals. Asterisks denote the significant differences between WT and AD mice: no significance (no asterisk), *p* ≥ 0.05; *p < 0.05; **p < 0.01; and ***p < 0.001.

Astrocyte reactivity is another hallmark of AD and part of the neuroinflammation process. A well-established marker for reactive astrocytes is the upregulation of GFAP (Leng and Edison, 2021). Therefore, we labeled GFAP in brain sections to characterize the astrogliosis in the central auditory pathway of AD mice. Astrocyte activation is evident in the AC of 5xFAD mice (Fig. 7*A*, top panel). The area covered by the GFAP signal was quantified and is presented in Figure 7*B*. In the AC, the increase of GFAP coverage was significant in 5xFAD mice compared with the WT controls (6M 5xFAD, *p* = 0.002; 12M 5xFAD, *p* = 0.002; Wilcoxon test). In the MGB, the increase of GFAP coverage was also significant in 5xFAD mice (6M 5xFAD, *p* = 0.002; 12M 5xFAD, *p* = 0.002; Wilcoxon test). In the IC, 5xFAD mice showed an increase of GFAP expression beginning at 6M (compared with WT; 6M 5xFAD, *p* = 0.002; 12M 5xFAD, *p* = 0.002). Overall, we conclude that astrocyte activation parallels the microglia activation in the auditory pathway of AD mouse models: it was significant in the IC, MGB, and AC of 5xFAD mice beginning at 6M.

### Mice showed the increased level of microglia coverage but not activation in the SOC and MNTB at 12M of age

**Figure 8.**
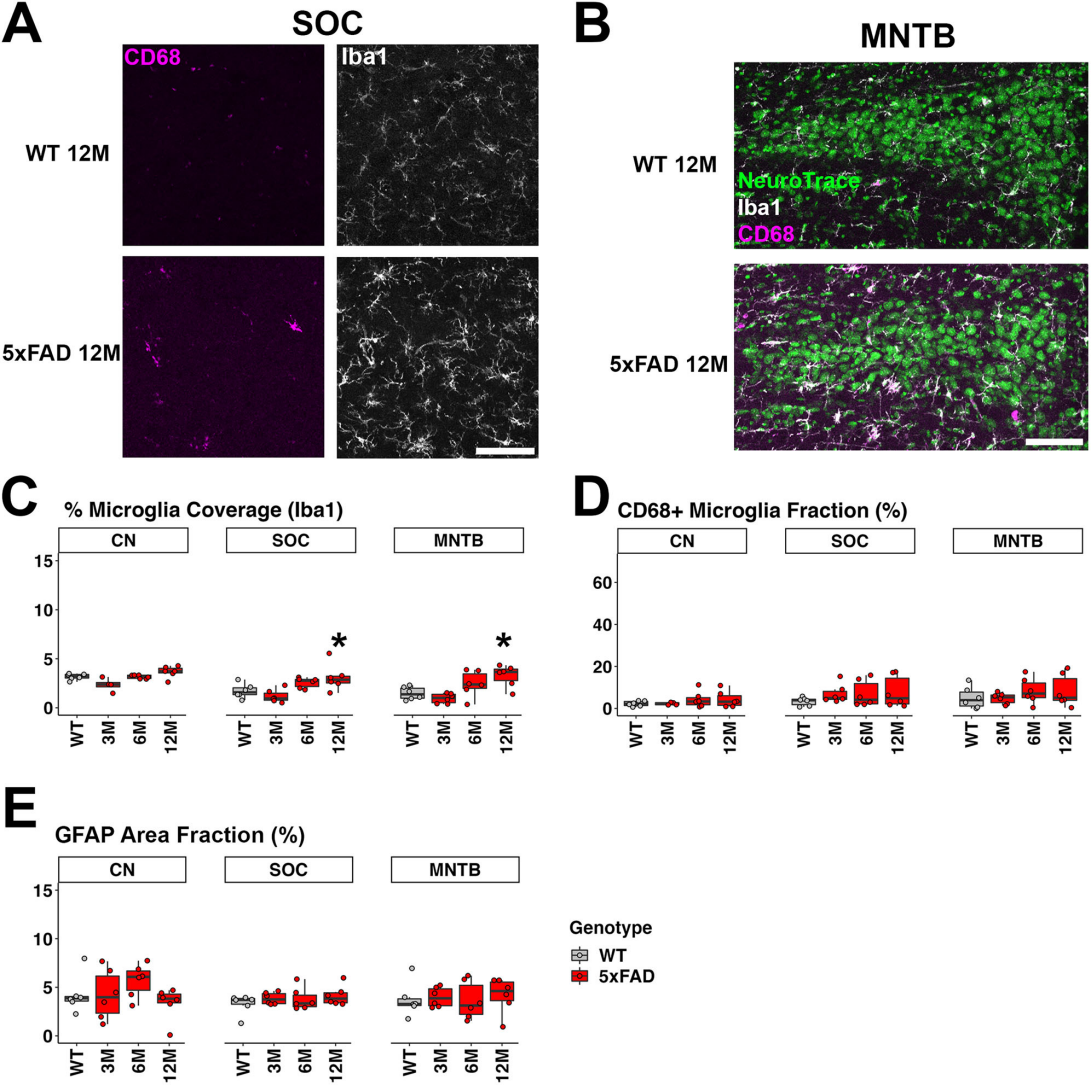
Neuroinflammation markers were absent from the brainstem areas in 5xFAD mice that are associated with the earliest stages of auditory processing. ***A***, Representative views of the SOC in 5xFAD and WT mice at 12M, with labels for Iba1 (white, right) and CD68 (magenta, left). ***B***, The MTNB (triangular shape of a neuron cluster) in 5xFAD and WT mice at 12M, with labels for NeuroTrace (labels neurons, green), Iba1 (white), and CD68 (magenta). The microglia coverage quantification (***C***), proportion of CD68+ microglia (***D***), and activated astrocyte coverage (***E***, GFAP Area Fraction) in the CN, SOC, and MNTB are also presented (*n* = 6 for each group). The genotypes for ***C***–***E*** are indicated in the legend of ***E***. Scale bar, 100 μm. Asterisks denote the significant differences between WT and 5xFAD mice: no significance (no asterisk), *p* ≥ 0.05; *p < 0.05; **p < 0.01; and ***p < 0.001.

Given the possible functionality change at lower levels of the auditory brainstem in 5xFAD mice at 12M, we examined microglia and astrocyte activation in the SOC, MNTB, and CN. We found that 5xFAD mice at 6 and 12M showed a higher mean of microglia coverage in the SOC and MNTB compared with WT mice (SOC, mean, 1.713% for WT 12M; mean, 2.568% for 5xFAD 6M; and mean, 3.094% for 5xFAD 12M; MNTB, mean, 1.474% for WT 12M; mean, 2.445% for 5xFAD 6M; and mean, 3.264% for 5xFAD 12M; Fig. 8*C*). The change is significant in 5xFAD mice at 12M of age (Fig. 8*A*–*C*; *p* = 0.041 for SOC; *p* = 0.015 for MNTB). However, the proportion of CD68+ microglia was not significantly increased in the SOC or MNTB of 5xFAD mice (Fig. 8*D*). Astrocyte activation was also not significant in the SOC or MNTB (Fig. 8*E*). In the CN, the percentage of microglia coverage, proportion of CD68+ microglia, and astrocyte activation were not significant in 5xFAD mice (Fig. 8*C*,*D*). Overall, our observations suggest increased microglia coverage in the SOC and MNTB of 5xFAD mice at 12M, but further analysis did not support an interpretation of glial activation.

### No changes of peripheral neuronal structures were detected

**Figure 9.**
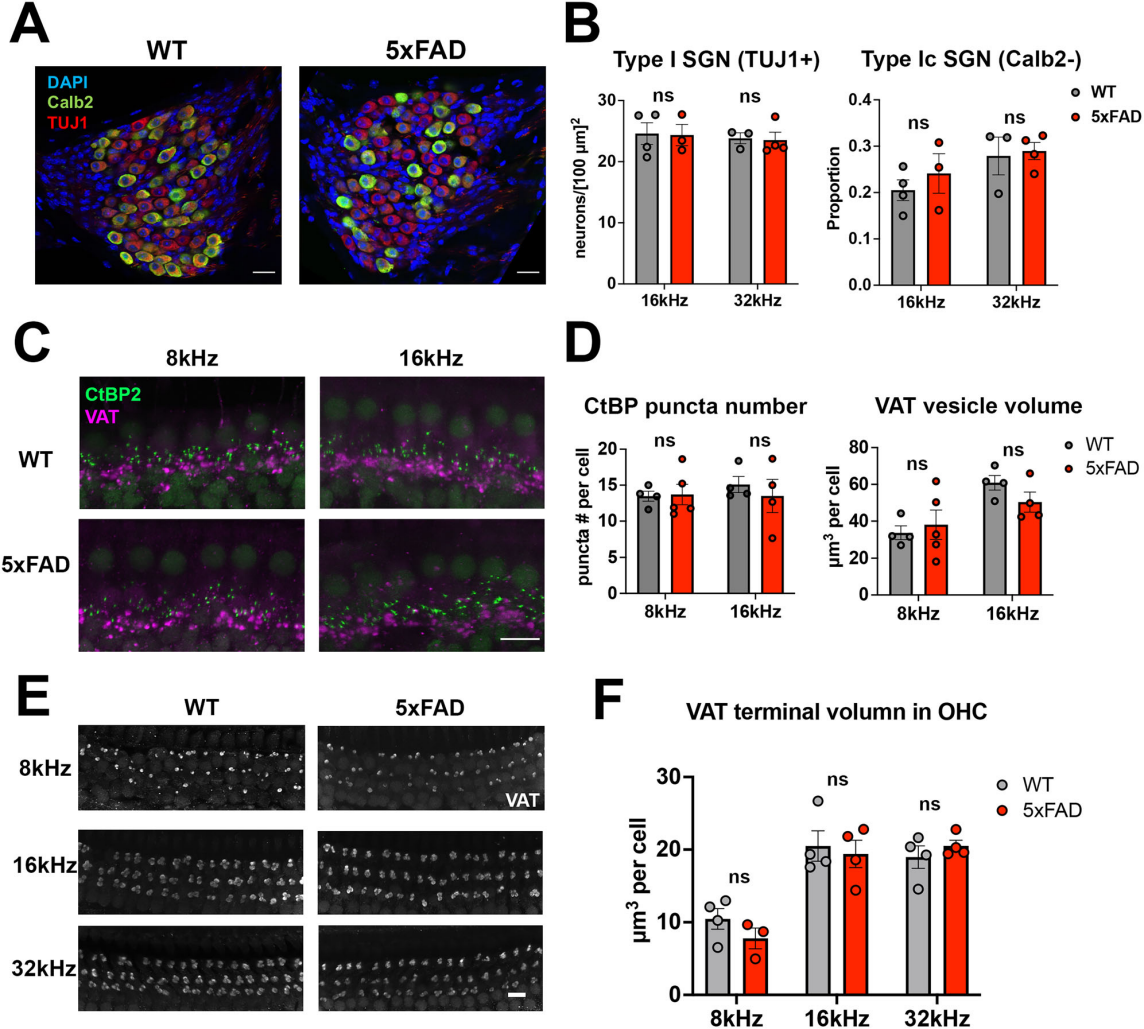
No differences in peripheral neuronal structures were observed between genotypes. ***A***, Representative view of SGN labeling in 5xFAD and WT mice at 12M. Images show the 16 kHz regions, with labels for DAPI (blue), Calb2 (green), and TUJ1 (red). ***B***, Quantification of Type 1 SGN density (left) and the proportion of Type 1c SGNs (right). ***C***, Representative view of afferent and efferent terminal labeling in cochlear whole mounts from 5xFAD and WT mice at 12M, with labels for CtBP2 (green) and VAT (magenta). Tonotopic cochlear regions for 8 kHz (left column) and 16 kHz (right column) are shown. ***D***, Quantification of CtBP2 puncta number per IHC (left) and mean VAT terminal volume per IHC (right). ***E***, Representative view of efferent terminal labeling (VAT in white color) in cochlear whole mounts from 5xFAD and WT mice at 12M. The 8 (top row), 16 (middle row), and 32 (bottom row) kHz regions are shown. ***F***, Quantification of VAT terminal volume (µm^3^) per OHC. Asterisks denote the significance of differences between WT and 5xFAD mice: no significance (ns), *p* ≥ 0.05; *p < 0.05; **p < 0.01; and ***p < 0.001, Student's *t* test or Wilcoxon test. Scale bar, 10 μm for ***A***, ***C***, and ***E***.

We previously observed an ABR Wave I amplitude reduction in 5xFAD mice at 12M (Na et al., 2023), which is generally considered a reflection of synapse loss or SGN degeneration in the cochlea (Kohrman et al., 2020). SGN and synapse loss are also a possible cause for central auditory hyperactivity (Auerbach et al., 2014; Schrode et al., 2018). We therefore examined the synapse loss and SGN degeneration in the cochleae of 5xFAD mice and their WT littermates at 12M (Fig. 9). TUJ1, the Type 1 SGN-specific marker (Sekerkova et al., 2008), was labeled in the spiral ganglions of cochleae. These neurons were colabeled with Calb2, a marker differentially expressed in subtypes of Type 1 SGNs (Fig. 9*A*; Shrestha et al., 2018). Type 1c SGNs, which are characterized as Calb2-negative neurons, are more vulnerable to insults and possibly the main source of the Wave I amplitude (Furman et al., 2013; Shrestha et al., 2018). The 16 kHz region was our primary focus because it showed the greatest change at the onset of hearing loss (9M) in 5xFAD mice (Na et al., 2023). As the 32 kHz region showed significant hearing loss in 5xFAD mice at 12M (Na et al., 2023), it was also included in this assay. 5xFAD mice did not show significant changes in either region (Type 1 SGN density, 16 kHz, *p* = 0.93; 32 kHz, *p* = 0.86; Type 1c proportion, 16 kHz, *p* = 0.45; 32 kHz, *p* > 0.99; unpaired Student's *t* tests and Wilcoxon tests; Fig. 9*B*).

Synapse loss, which is considered an event prior to SGN loss (Kujawa and Liberman, 2009), was further examined in those mice. CtBP2, the presynaptic ribbon marker, was labeled in whole-mount cochleae to characterize afferent synapse loss (Fig. 9*C*). The CtBP2 number of puncta per IHC was quantified (Fig. 9*D*). The 8 and 16 kHz regions were primarily focused given that those regions showed significant difference at the onset of hearing loss in 5xFAD mice. Synapse loss was not significant in either region in those mice (8 kHz, *p* = 1; 16 kHz, *p* = 0.89; Wilcoxon tests).

Cholinergic lateral olivocochlear (LOC) efferents, which innervate the auditory nerve dendrites beneath the IHCs, regulate auditory nerve activity and potentially protect the auditory nerve (Wu et al., 2020; Lauer et al., 2022). Therefore, we also examined the LOC efferent terminals by labeling with VAT (Fig. 9*C*). Individual terminals were indistinguishable with the current resolution, and therefore the average VAT-positive terminal volume was quantified (Fig. 9*D*, right graph). 5xFAD mice did not show a significant loss of LOC efferents in 8 and 16 kHz regions (8 kHz, *p* = 0.72; 16 kHz, *p* = 0.43; unpaired Student's *t* tests).

The medial olivocochlear (MOC) efferents, which innervate the OHCs, protect hair cells from aging and noise damage (Maison et al., 2013; Liberman et al., 2014). The MOC efferents have also been proposed to contribute to hearing in noise (Lauer et al., 2022), in which the 5xFAD mice showed significant amyloid accumulation (Na et al., 2023). Therefore, we examined the MOC efferent terminals which were labeled by VAT in OHC regions (Fig. 9*E*). The quantification of the average VAT terminal volume per hair cell is shown in Figure 9*F*. 5xFAD mice did not show a significant change in the 8, 16, or 32 kHz regions (8 kHz, *p* = 0.25; 16 kHz, *p* = 0.71; 32 kHz, *p* = 0.40; unpaired Student's *t* tests).

Overall, no significant SGN, synapse, and/or efferent losses were found in 5xFAD mice at 12M. We conclude that diagnostic ABR changes shown in Figure 2 are not due to peripheral structural losses.

## Discussion

Here we evaluated whether differences in the ABR, a simplified auditory event-related potential, can be used to identify amyloidosis in the transgenic mouse model 5xFAD with machine learning classification. The classification produced 79% accuracy in 5xFAD mice at 6M and 89% accuracy at 12M. Notably, 6M is also the age at which 5xFAD mice become significantly less responsive to a cue after fear conditioning. Further interpretation of the machine learning classification suggests that the activity change was pronounced in the upper auditory brainstem of 5xFAD mice at 6M. Histological examination revealed significant microglia and astrocyte activation in nuclei of the upper auditory brainstem, including the IC and MGB, consistent with the interpretation of machine learning classification. In addition, no correlation was observed between changes in peripheral structures and central auditory hyperactivity. Taken together, this evidence supports the hypothesis that auditory processing analysis can assist the diagnosis of early-stage AD.

### Machine learning can reliably distinguish the ABR waveforms of 5xFAD mice from WT littermates

Advances in artificial intelligence are growing at a remarkable rate due to the rapid development of machine learning algorithms. The power of machine learning algorithms lies in their ability to capture subtle changes and handle high-dimensional data (i.e., the dataset with the number of features/variables close to or larger than the number of observations). Machine learning algorithms have been widely applied to disease diagnostics in combination with approaches yielding results with complex features, such as imaging or Raman spectroscopy (Latif et al., 2019; Qi et al., 2023). In the present study, using a machine learning algorithm, an ABR abnormality was able to diagnose AD in 5xFAD mice when AD hallmarks were distributed to the auditory brainstem. It should be noted that the diagnosis was carried out using the ABR recordings evoked by click at only one sound level (65 dB), which can be acquired in minutes. We propose ABR as a low-cost, noninvasive, and rapid approach to assist diagnosis of early-stage AD with the power of machine learning. However, as many other studies on AD, this study is limited by the use of the AD transgenic model, which can only partially reflect the human AD pathophysiology of AD. For this reason, we expect that if ABR analysis has utility in tracking changes within the upper auditory brainstem of AD patients, it will only be for a subset. However, that in itself could be interesting if ABR analysis can reliably stratify AD patients at early stages.

The interpretability of some machine learning algorithms can facilitate the identification of changing features, which has been applied in explaining diagnostic results such as Raman spectrum from AD brain tissues (Wang et al., 2022). In the present study, the machine learning algorithm identified the salient features of ABR that were changing in 5xFAD mice. Disruption to those specific ABR generators was further confirmed by histological analysis. This result suggests that the application of this approach can identify functionality changes of the auditory brainstem in AD patients at different stages or with differential AD hallmark distributions. In this approach, we preferentially use data from click stimuli, which are short presentations of broadband sounds. There is evidence that older patients display variability in their later ABR waveforms in response to clicks or chirps (Maass et al., 2024), and clicks can be employed with subjects who have high-frequency hearing loss. We note that advanced approaches such as parallel ABR can obtain similar recordings in humans at multiple frequencies up to six times faster (Polonenko and Maddox, 2019). Their use may enable researchers to rapidly obtain more comprehensive information about ABR changes in human patients. Our results suggest that with the power of machine learning algorithm, ABR assessment could help researchers acquire new insights into neuropathology changes in AD.

### Neuroinflammation as a link between central auditory hyperactivity and AD

In the present study, we characterized microglia and astrocyte activation in the auditory brainstem of amyloidosis mice over time. Previously, we had documented significant Aβ plaque deposition in the AC, MGB, IC, and SOC of 6M 5xFAD mice (Na et al., 2023). Here we found that Aβ plaque deposition is sufficient to induce reactive gliosis only in the AC, MGB, and IC. Reactive gliosis is the key to understanding the central auditory hyperactivity in 5xFAD mice, which results from an imbalance of excitatory and inhibitory signaling (Auerbach et al., 2014). The upregulation of proinflammatory factor, TNF-α, is currently the only known factor able to mediate this imbalance in the auditory system (Wang et al., 2019; Shulman et al., 2021). It is likely to happen in the regions where microglia activation is triggered by Aβ plaque deposition (Leng and Edison, 2021). Further investigation is necessary to better understand this correlation. Assessing preconscious auditory processing, inflammation, and AD hallmarks in the upper auditory brainstem in MCI and early AD patients will be key to relating these findings to human health. We speculate that the onset of neuroinflammation and hyperactivity in the IC will have an adverse effect on normal preconscious integration of visual and auditory information (Schmehl and Groh, 2021), driving confusion and further decline in patients.

### Behavioral changes in 5xFAD mice reported here are similar to previous reports

To determine if the development of the ABR abnormality coincides with memory deficits, we performed behavioral assays to evaluate 5xFAD mice at different ages. 5xFAD mice have been reported to develop a number of age-related neural deficits within the time course of our observation (3–12M), including memory impairment (4–5M), reduced emotionality/anxiety (6M), motor deficits (9M), and recognition impairment (10M; Oakley et al., 2006; Jawhar et al., 2012; Locci et al., 2021). 5xFAD mice were reported to show reduced anxiety starting at 6M (Jawhar et al., 2012) in the elevated-plus maze test. When anxiety was measured using the open-field test, results varied. In some studies, 5xFAD mice spent more time in the center zone at 7M (Belaya et al., 2020) or 8M (Forner et al., 2021), but not at 12M or later (Forner et al., 2021), suggesting temporarily reduced anxiety in those mice. Other studies showed that the 5xFAD mice tended to explore the center less at 10 or 11–13M (Locci et al., 2021; O'Leary and Brown, 2022). We saw no change in open-field behavior in 5xFAD mice at 3, 6, or 12M.

In the present study, memory impairment was evaluated by a fear conditioning test. Previous studies reported that 5xFAD mice showed significantly worse performance in a contextual fear conditioning test beginning at 6M (Ohno, 2009). However, in our hands, this test did not reveal significant differences between 5xFAD mice and their WT littermates at any age. Similarly, a more recent study, which performed comprehensive phenotyping of 5xFAD mice (Forner et al., 2021), found insignificant performance changes in 5xFAD mice up to 12M. Our results are more consistent with those of Forner and colleagues. In contrast to contextual fear, we did find that 5xFAD mice performed worse at 6 and 12M in the cued fear conditioning test. This is consistent with previous studies (Bouter et al., 2014; Yang et al., 2017). The difference in freezing scores between the novel session and cue test session demonstrated that 5xFAD mice were successfully trained. As ABR thresholds are not different at 6M between 5xFAD and WT littermates (Na et al., 2023), we conclude that impaired performance of those mice in this test was not due to an inability to detect the auditory cue, which was presented at 80 dB.

Collectively, with this set of behavioral assays, we observed significant behavioral changes in 5xFAD mice only in the cued fear conditioning test. Overall, we conclude that the behavioral changes of 5xFAD mice in the cued fear condition test are consistent with some previous studies, not due to a measurable increase in anxiety or loss of locomotion, and independent of hearing loss. Moreover, these changes arise at the same time as the onset of central auditory hyperactivity, suggesting the latter may be useful as an independent biomarker for the former. However, we note that mouse models have significant limitations compared with studying neurocognitive phenomena in humans, necessitating further research.

### Caveats and future directions

Several factors pose barriers to the translation of information in the present study to improved diagnostic testing in human presymptomatic or early-stage AD. This study is limited by the use of the 5xFAD transgenic AD model. While this model mimics many attributes of human AD, human AD pathophysiology is only partially understood. No mouse model cannot fully represent all the features of AD. However, our approach showed great consistency between machine learning feature interpretation and amyloid plaque distribution (Figs. 2*D*, 3), suggesting its power to indicate the AD hallmark distribution in the brain. The efficiency of using ABR to assist AD diagnosis needs further investigation, especially in AD humans with different subtypes and different AD hallmark distribution.

In addition, approach presented in this study needs elaborate methods to accurately discriminate between mutant and control animals’ response to a simple click signal, which presents a limitation for improving diagnostic testing. This approach may be improved in future studies, by testing ABRs from multiple sound levels and frequencies, as well as from humans/mouse models with different AD symptoms.

## Conclusion

Overall, we conclude that neuroinflammation, which has been proposed as the link between AD and central auditory hyperactivity in 5xFAD mice, temporally and spatially correlates with central auditory hyperactivity in those mice. The reliable performance of 5xFAD mouse identification using ABR data and machine learning suggests that further research into this approach for AD diagnosis may help some patients. We suggest further investigation into AD-driven pathology in the thalamus and midbrain of human patients, as damage to these structures may correlate with the transition from MCI to overt dementia.

## Data Availability

The datasets used and/or analyzed during the current study are available from the corresponding author on request. The datasets generated and/or analyzed during the current study are available in the Open Science Framework repository.

## Synthesis

Reviewing Editor: Harald Sontheimer, University of Virginia Decisions are customarily a result of the Reviewing Editor and the peer reviewers coming together and discussing their recommendations until a consensus is reached. When revisions are invited, a fact-based synthesis statement explaining their decision and outlining what is needed to prepare a revision will be listed below. The following reviewer(s) agreed to reveal their identity: John Breitner, Jung-Bum Shin. Note: If this manuscript was transferred from JNeurosci and a decision was made to accept the manuscript without peer review, a brief statement to this effect will instead be what is listed below.

The two referees raise some concerns that I believe can be addressed by carefully revising and adding to the discussion the caveats that are intrinsic to mouse models of AD. Also, it appears prudent to tone down the translatability to human disease i.e. the development of human diagnostic. The mouse work, however is deemed very interesting and carefully executed. I am providing significant comments from both referees that may guide the authors to a careful editorial revision.

"A strength of this paper is its comprehensive integration of electrophysiological (ABR), neuroinflammatory (microglia and astrocyte activation), and behavioral (auditory fear conditioning) analyses, providing a holistic understanding of auditory deficits in AD. The quality of the data is appropriate, and no additional experiments are requested, but limitations of this approach should be elaborated in the Discussion section:"

"there are two substantial limitations here to this approach. One is the verisimilitude of the 5X Tg model as a substitute for human AD. This model does mimic many of the known attributes of human AD, but the pathophysiology of human AD is only partially understood, and it's uncertain that the model accurately represents all the (known and unknown) features of this disease. Second is the limitations of mouse models for studying neurocognitive phenomena in humans. Here it seemed necessary to use very elaborate methods to discriminate accurately between mutant and control animals' response to a simple click signal. These two concerns pose threats to the translation of information here to improved diagnostic testing in human pre symptomatic or early-stage AD. In my opinion, the authors need to re-write their paper to deal more openly with these sorts of concerns."

## Author Response

Author's rebuttal Synthesis Statement for Author (Required):

The two referees raise some concerns that I believe can be addressed by carefully revising and adding to the discussion the caveats that are intrinsic to mouse models of AD. Also, it appears prudent to tone down the translatability to human disease i.e. the development of human diagnostic. The mouse work, however is deemed very interesting and carefully executed. I am providing significant comments from both referees that may guide the authors to a careful editorial revision. "A strength of this paper is its comprehensive integration of electrophysiological (ABR), neuroinflammatory (microglia and astrocyte activation), and behavioral (auditory fear conditioning) analyses, providing a holistic understanding of auditory deficits in AD. The quality of the data is appropriate, and no additional experiments are requested, but limitations of this approach should be elaborated in the Discussion section:" "there are two substantial limitations here to this approach. One is the verisimilitude of the 5X Tg model as a substitute for human AD. This model does mimic many of the known attributes of human AD, but the pathophysiology of human AD is only partially understood, and it's uncertain that the model accurately represents all the (known and unknown) features of this disease. Second is the limitations of mouse models for studying neurocognitive phenomena in humans. Here it seemed necessary to use very elaborate methods to discriminate accurately between mutant and control animals' response to a simple click signal. These two concerns pose threats to the translation of information here to improved diagnostic testing in human pre symptomatic or early-stage AD. In my opinion, the authors need to re-write their paper to deal more openly with these sorts of concerns." We agree with the reviewer and have made the requested changes.
